# Ocular Surface Diseases in Patients With Diabetes

**DOI:** 10.7759/cureus.23401

**Published:** 2022-03-22

**Authors:** Kunj Naik, Renu Magdum, Amod Ahuja, Sucheta Kaul, Johnson S, Ashish Mishra, Mayur Patil, Dr. Nilay Dhore, Aparna Alapati

**Affiliations:** 1 Ophthalmology, Dr. D. Y. Patil Medical College, Hospital and Research Centre, Pune, IND; 2 Ophthalmology, LV Prasad Eye Institute, Hyderabad, IND; 3 Community Medicine, Dr.D.Y.Patil Medical College, Hospital and Research Centre, Pune, IND

**Keywords:** glycated hemoglobin (hba1c), osdi scoring system, meibomian gland disorders, dry eye, tear film

## Abstract

Purpose

Diabetes is a major cause of ocular morbidity as multiple mechanisms play a role in inducing inflammatory changes in the eye. Diabetic retinopathy is the most common complication and is well-documented. However, in the era of modern medicine, attention is also being focused on ocular surface changes in diabetes. Therefore, this study aimed to determine the association between diabetes and ocular surface diseases.

Materials and Methods

This is a cross-sectional study examining 320 eyes of 160 patients with diabetes who were grouped according to their duration of diabetes. The symptoms were evaluated using the ocular surface disease index (OSDI) questionnaire. Their recent hemoglobin (Hb) A1c value was recorded. Their external or internal hordeolum, blepharitis, meibomian gland dysfunction, and corneal sensitivity were also evaluated. The tear film was examined using tests, such as Schirmer's test, tear film breakup time (TBUT), tear film meniscus height (TFMH), fluorescein stain, and rose bengal stain. The results were correlated with the duration and control of diabetes.

Results

The mean age of the study population was 56.60 years comprising 56% (n=89) females and 44% (n=71) males. The mean OSDI scores were 7.9 ± 3.55 and 57 ± 19.22 in patients without dry eye and with severe dry eye, respectively. The study observed OSDI scores were consistently high with diabetes severity. About 67% (n=24) of patients with HbA1c of >8% had dry eyes. Dry eye was found in 68% (n=59) of patients with the duration of diabetes being >10 years. About 23.7% (n=38) had blepharitis, whereas only 4% (n=7) had external or internal hordeolum and 44% (n=86) had different grades of meibomian gland dysfunction. Corneal sensitivity was abnormal in only 12% (n=12) of patients. About 55% (n=86) of patients had varying degrees of dry eye. A statistically significant correlation was found between the severity of dry eye and TBUT, TFMH values, and grades of corneal staining (P < 0.0001).

Conclusion

This study observed that the incidence of dry eyes was found to be higher when patients had uncontrolled diabetes and diabetes for a longer period. The OSDI scoring system is an important diagnostic tool while examining patients with dry eye. In an ophthalmology clinic, patients with diabetes should always be evaluated for any ocular surface changes when being screened for diabetic retinopathy, and proper guidelines should be implemented to detect changes in the ocular surface system as early as possible so that any long-term complications such as infectious or neurotrophic keratitis may be avoided at an early stage.

## Introduction

Next to China, India has 69.2 million patients with diabetes mellitus (DM), the second-highest number worldwide [1]. According to reports, DM levels are estimated to be 12% to 15% in India [2]. Among people aged between 20 to 70 years, DM is one of the major causes of preventable blindness. A person with blindness caused by DM may experience complications such as neuropathy, retinopathy, nephropathy, and cardiovascular disorders [3]. As for the eye, diabetic retinopathy has been well studied and documented. However, ocular surface diseases in DM have gained some significance as it hampers patients’ quality of life [3].

“The ocular surface complex” is defined as the ocular surface, including the corneal surface with its epithelium, conjunctiva, and its superficial layers and the ocular adnexal system containing the lacrimal, meibomian gland, and eyelashes, among others, and eyelid with its blinking mechanism. The diseases associated with these structures are known as “ocular surface diseases” [4]. Among the ocular surface diseases, dry eye syndrome is the most common. Multiple mechanisms, such as ocular surface and lacrimal gland inflammation, neurotrophic deficiency, and meibomian gland dysfunction (MGD), play significant roles. It had been suggested that one or more of the following initial events may lead to alterations described in the tear film and ocular surface of patients with DM: a) chronic hyperglycemia, b) corneal nerve damage, and c) impairment on insulin action [5].

In India, the prevalence of ocular surface diseases ranges from 17% to 69%. The reported percentages of ocular surface disorders and dry eye have been approximately 54% [6]. Ocular surface diseases in DM are quite common among patients with DM. However, understanding of symptoms is unfortunately lacking, and assessment errors result in many undiagnosed or undertreated patients. As most data on diabetic ocular surface disease are based on studies from Western Europe, and since DM incidence in India has been increasing, the pattern and knowledge in Indian populations should also be examined. Hence, this study is conducted with the Indian population in consideration.

## Materials and methods

This cross-sectional study was conducted from September 2019 to September 2021 at Dr. D. Y. Patil Medical College, Hospital & Research Center, a tertiary care hospital in Pimpri, Pune, India. This study was conducted in the outpatient ophthalmology department that examined 320 eyes of 160 consecutive patients with DM regardless of their age, gender, duration of disease, and type of DM. Patients with pterygium and thyroid disease, those who used chronic ocular drugs such as anti-histamines (olopatadine, fexofenadine etc.), wore contact lenses, had corneal opacity due to trauma, or those who underwent ocular surgery in the past three months, and are on anti-depressants (citalopram, fluoxetine etc.), were excluded. Ethical committee clearance was obtained from the Institutional-Ethics Sub-committee at Dr. D. Y. Patil Medical College, Hospital and Research Centre, Pimpri, Pune, India (Research Protocol No. IESC/PGS/2019/109). The study purpose was explained to all patients, and consent was obtained.

A detailed systemic illness history was taken. The best corrective visual acuity was noted. A recent result of the average sugar level with hemoglobin (Hb) A1c level was also recorded. Patients were evaluated after taking full history for symptoms, such as itching, dryness, grittiness, foreign body sensations, sticky eye discharges, redness, or any insensitivity to bright or low light using the ocular surface disease index (OSDI) questionnaire, and the total score was recorded. Detailed ocular examination was performed using slit-lamp examination with special attention given to the eyelash, eyelids, conjunctiva, and status of cornea. Eyelashes of both eyes were examined for any hard scales, crusting, dandruff-like scaling material, or presence of hyperemia of anterior lid margins. Eyelids were examined for any infections like external or internal hordeolum. The corneal sensations were checked using a cotton wisp. If instant blinking reflex was present, corneal sensations were noted as intact, and if the instant blink reflex was absent, then it was noted as abnormal corneal sensations.

The eyelids were also examined for the presence of any MGD by gently pressing the fingers on the lid margin to evaluate the expressed secretions for the expression and quality [7] (Table 1).

**Table 1:** The classification of meibomian glands based on quality and expressivity

Grades	Quality and expressivity of the meibomian glands
Grade 0	Absence of any abnormality
Grade 1	On lid margin compression, the presence of plugging with translucent serous secretion
Grade 2	On lid margin compression, the presence of plugging with viscous or waxy white secretions
Grade 3	On lid margin compression, absence of secretions

To evaluate patients’ basic and reflex tearing, a Schirmer’s test I without using topical anesthesia was performed with the standardized no.41. Whatman filter paper. After asking the patient to look up, the strips were placed at the lower fornix at the junction of lateral one-third and medial two-thirds of the eyelid while ensuring that it was not touching the corneal surface. The strip was put in place for five minutes, and the wet distance was measured in millimetres (mm). A reading of >10 mm was considered normal tearing, whereas a reading of <10 mm was considered poor tearing. If poor tearing was observed on Schirmer’s test I, the test was repeated with nasal stimulation. If the reading is >5 mm, it was noted­ as good reflex tearing, whereas if the reading is <5 mm, it will be noted as abnormal reflex tearing [8,9].

The TFMH was measured using a slit lamp, and 0.3 mm was set as a cut-off, and if the noted measurement was <0.3 mm, it was considered abnormal. The TBUT was examined on the slit lamp using a fluorescein strip using topical anesthesia. Using a timer, the visualization of the first dry spot from the last blink was noted. A TBUT reading of <10 s was noted as abnormal [8,9].

The staining pattern of the cornea was evaluated using a fluorescein strip containing 1 mg of fluorescein sodium [10,11] (Table 2, 3).

**Table 2:** The classification of staining density of the corneal surface area

Grades	Staining of the corneal surface area
Grade 0	No punctate staining
Grade 1	Less than 1/3^rd^
Grade 2	1/3^rd^ to 2/3^rd^
Grade 3	>2/3^rd^

**Table 3:** The classification of the staining density of the cornea

Grades	Staining density of the cornea
Grade 0	Absence of punctate staining
Grade 1	Representing sparse density
Grade 2	Representing moderate density
Grade 3	High-density overlapping regions

Both eyes were stained with rose bengal strip containing 1.5 mg of Rose Bengal to evaluate the conjunctival staining pattern. The nasal and temporal parts of the conjunctiva were stained using the Rose Bengal strip and graded as indicated in previous studies [12] (Table 4). The total of these staining scores of >3 was noted irregular.

**Table 4:** The classification of conjunctival staining pattern

Grades	Conjunctival staining pattern [12]
Grade 1	Absence of staining
Grade 2	Staining of very few points
Grade 3	Scattered staining pattern
Grade 4	Confluent areas of the stained conjunctiva

Statistical analysis

Data collected from the patients were coded and further tabulated. All data analyses were performed using Spearman’s rank correlation, Kruskal-Wallis tests and Statistical Package for the Social Sciences (SPSS) version 22 (IBM Corp., Armonk, NY) to find a statistical correlation between two variables.

## Results

In the study, patients consisted of 56% (n=89) females and 44% (n=71) males. The mean age of patients was 56.60 years, predominantly consisting of patients aged >60 years. About 37% (n=60) of patients were >60 years, whereas 10% (n=15) were 30 to 40 years, 21% (n=33) were 41 to 50 years, and 32% (n=52) were 51 to 60 years. About 45% (n=72) of patients had no dry eye, whereas 55% (n=88) were diagnosed with dry eye. Around 39% [n=62] of patients had mild dryness of eyes, 9% (n=15) had moderate dryness, and 7% (n=7) had severe dry eyes.

In this study, 54% (n=86) of patients had been diagnosed with DM for five to 10 years, 14% (n=23) had it <2 years, 17% (n=17) had it between two to five years, and 15% (n=24) patients had it for >10 years. A significant positive correlation was observed using Spearman’s rank correlation with a p-value of 0.405 and P <0.0001 between the duration of DM and dry eye (Table 5).

**Table 5:** Association of the duration of diabetes with dry eye syndrome

Duration of diabetes	Number of patients	Percentage (%)	Dry eye (%)
<2 years	23	14 %	4%
2–5 years	27	17%	7 %
5–10 years	86	54%	21%
>10 years	24	15%	68%
Total	160		

In our study, 29% (n=47) patients had HbA1c level of <6.5%, whereas 30% (n=48) had 6.5% to 8%, and 41% (n=65) had >8%. Dry eyes were observed in 67% (n=59) of patients with an HbA1c level of >8 (Table 6). A significant positive correlation was observed between HbA1c level and dry eye using Spearman’s rank correlation with p-value of 0.510 and P <0.0001.

**Table 6:** Association of HbA1c level and dry eye

HbA1c level	% of patients with dry eye (n=88)
<6.5	7
6.5–8	24
>8	67

The mean OSDI score was 7.9 ± 3.55 in patients without dry eyes, 21 ± 2.99 in mild dry eyes, 29 ± 2.89 in moderate dry eyes, and 57 ± 19.22 in severely dry eyes. Only seven of 160 patients had external or internal hordeolum, whereas 23.7% (n=38) of patients had blepharitis. In this study, 44% (n=71) patients had Grade 0 MGD; 39% (n=63) had Grade 1; 9% (n=14) had Grade 2; and 8% (n=12) had Grade 3. Comparing these results with dry eye, a statistically significant positive correlation was observed between the severity of dry eye and MGD grades (P < 0.0001 and p-value 0.340). Low Schimer’s test, TFMH test, and TBUT scores were noted. The study found the mean TFMH in patients with no dry eye to be 0.7 ± 1.96 mm, and 0.2 ± 1.38 mm in patients with dry eyes, suggesting a statistically highly significant correlation (using Kruskal-Walis test) with the severity of dry eye as compared to non-dry eye patients in the TFMH test (P < 0.0001).

The TBUT value was 14 ± 1.56 (s) and 8.92 ± 1.32 (s) in patients without and with dry eyes, respectively, suggesting a statistically highly significant correlation (Kruskal-Wallis test) with the severity of dry eye as compared to patients without dry eyes in the TBUT test (P < 0.001). The mean corneal staining grading was 0.64 ± 0.32 and 1.43 ± 0.87 in patients without and with dry eyes, respectively, which was observed to have a high statistical significance when correlated with the dry eye patients as compared to patients without dry eyes using the Kruskal-Wallis test (P < 0.0001).

In our study, the mean grading of corneal staining density was seen to have a statistically highly significant correlation using the Kruskal-Wallis test, with patients with dry eye (1.46 ± 0.21) as compared to patients without dry eyes (0.64 ± 0.69) (P < 0.0001). About 22% of patients had varying degrees of conjunctival staining: 11% (n=17) had Grade 1; 9% (n=14) had Grade 2; and only 2% (n=4) had Grade 3. In our study, 12% (n=20) of patients had abnormal corneal sensation, whereas 88% (n=140) of patients had intact.

## Discussion

This study aimed to investigate the ocular surface disease in patients with DM. This study focused to find a positive correlation between ocular surface disorders and DM. Khetwani et al. reported that 148 of their 182 patients were aged >50 years [13]. Similarly, Sarkar et al. reported the mean age of patients was 54 ± 10.06 years with 43% aged between 40 and 50 years [14]. Acharlu et al. demonstrated that the mean age of patients with DM was 55.06 ± 9.70 (range 40-75) years [15]. Khetwani et al. [13] categorized their patients into multiple groups as per the duration of DM.

The calculated HbA1c value of the study showed that dry eyes were more common in patients with DM who had abnormal levels (52%) than in those with normal levels. A study conducted by Dutta et al. in Assam, India, observed a statistically significant correlation when comparing Schirmer’s score and the HbA1c level of each patient [16]. Sarkar et al.’s study in West Bengal, India, showed that DM management was highly statistically significant with dry eyes [14]. Patients with poor glycemic control (HbA1c >8%) were found to have a higher degree of dry eyes. Kaiserman et al. [17] and Seifart et al. [18] reported a positive association between the DM control using HbA1c values and the dry eye syndrome.

A significant positive correlation was observed between the duration of DM and the occurrence of dry eyes. A similar observation was reported by He et al. [19], Yoon et al. [20], and Manaviat et al. [6]. The duration of DM was significantly associated with dry eyes (P = 0.01) according to Khetwani et al. [13] with statistical significance (P = 0.04). Nasar et al. [21] similarly observed a significant association of dry eyes with poor glycemic control (P < 0.001) and with a longer duration of DM (P < 0.05) in a study conducted in Tamil Nadu, India. Gannur et al. [22] in Vijaypura district, Karnataka, India, observed that dry eyes were more common in patients with five years of DM (a 2.65-fold increase). A highly statistically value of P < 0.001 was observed in patients with dry eyes who had uncontrolled DM.

In our present study, the mean OSDI score was 7.9 ± 3.55, 21 ± 2.99, 29 ± 2.89, and 57 ± 19.22 in patients without, mild, moderate, and severe dry eyes, respectively. The grades of dry eye were significantly associated with high OSDI scores. Fuerst et al. [23] reported a significant correlation between the tear-film osmolarity score and OSDI score among patients with DM. Schiffman et al. [24] evaluated the usefulness of the OSDI scoring system in 109 participants with dry eyes and 30 healthy participants, and conclude this questionnaire is a valid and reliable instrument for calculating the severity of dry eye disease. Khetwani et al. [13] and Zhang et al. [25] observed similar results. A study conducted by Kalaivani et al. [26] in 2017 in Puducherry, India among 191 patients with DM showed the prevalence of dry eye was 51.8%. About 42% prevalence of dry eye was reported by Sarkar et al. [14] and 54% by Manaviat et al. [6].

Our study showed the mean value of TFMH was 0.7 ± 1.96 (mm) in patients without dry eyes and 0.2 ± 1.38 (mm) in patients with dry eyes. Our study noted that the mean TFMH statistically, highly, and significantly correlated with the severity of dry eyes as compared to those without dry eyes (P < 0.005). Keserwani et al. [27] observed significantly lower Schirmer’s score and TBUT measurements in patients with DM than those without (P < 0.001). Patil et al. [28] also reported that patients with DM aged 50 years were examined for dry eye tests and observed a statistically significant association between the duration of DM and dry eyes (P = 0.00). Low Schimer’s test (48%) and TBUT (68%) values were found in patients with DM. Some amount of dryness was present in 34 subjects (68%). Similar observations were present in studies done by Sarkar et al. [14], Wasir et al. [22], and Kamel et al. [29].

In our study, blepharitis was observed in 23.7% of patients, whereas MGD was observed in 53.75% of patients. Lee et al. [30] stated that blepharitis is significantly related to metabolic syndromes such as DM, and can serve as an early sign of DM. Hom et al. [31] and Ansari et al. [32] observed similar results in their studies. In our present study, 44% of patients had Grade 0 MGD, 39% had Grade 1, 9% had Grade 2, and 8% had Grade 3, suggesting a positive correlation between the severity of dry eyes and grades of MGD. Manjula et al. [33] found 56% of patients in their study had dry eyes, and 24 of them had MGD. Shamsheer et al. [34] found that MGD was more common in the diabetic group (15%) compared to only 7% found in the control group with a p-value of 0.0015. Kumar et al. [7] found that 50% had MGD along with 54% showing varying degrees of dry eyes, which was statistically significant (P = 0.001). Kesarwani et al. [26] observed that when diabetics were compared with healthy controls, the diabetic group showed higher grades of corneal staining (P < 0.001) and conjunctival staining (P < 0.001).

Limitations

The study population was not followed up to record any disease progression or improvements. Considering a higher number of patients in the country, the sample size of 320 patients is inadequate. In the study, occupations and social activities such as reading or screen time on electronic devices were not taken into account as they can also cause dry eyes. The newer diagnostic tests such as confocal microscopy for corneal neuropathy, tear film osmolality, use of optical coherence tomography, refractive meniscometry for TFMH measurement, infrared meibography, interferometry to analyze lipid layer of the tear film, analysis of biomarkers in the tear film, the tear film stability analysis system (videokeratography), and ocular surface thermography were not implemented in the study although the outpatient department was equipped with these instruments.

## Conclusions

Long-term DM predisposes to changes in the tear film causing ocular surface irregularities, which lead to dry eyes. Dry eye is common in patients with diabetes, and the same was proven in our study. The use of the OSDI scoring system is justified in this study. The OSDI questionnaire is an integral part of the examination, and its use should be a benchmark while diagnosing a patient with OSDs as its validity and reliability are unquestionable. The duration of DM plays an important role in ocular surface diseases. This study observed that dry eyes more frequently occur when patients have DM for a longer period. In an ophthalmology clinic, patients with DM should always be evaluated for any ocular surface changes when being screened for diabetic retinopathy, and proper guidelines should be implemented to detect changes in the ocular surface system as early as possible so that any long-term complications may be avoided at an early stage.
